# Research on the correlation between the processing technology of conjugated nanomaterials and the design of sports equipment

**DOI:** 10.3389/fchem.2023.1327618

**Published:** 2023-12-11

**Authors:** Yaguo Miao

**Affiliations:** School of Physical Education, Zhengzhou Normal University, Zhengzhou, Henan, China

**Keywords:** nanomaterials processing technology, sports equipment design, carbon nanotubes, principles of correlation, conjugated nanomaterials

## Abstract

The processing technology of conjugated nanomaterials and the design of sports equipment are often regarded as two independent fields, resulting in poor performance in sports equipment design. Analyze the characteristics, preparation methods, and potential applications of conjugated nanomaterials, and use the unique properties of conjugated nanomaterials to improve the performance of sports equipment. Graphene conducts electricity very well and is the hardest among nanomaterials. Carbon Nanotubes (CNTs) exhibit good thermal conductivity. Therefore, this paper proposes to use CNT and graphene materials in Natural Rubber (NR) composites in sports equipment, and explores the relationship between the two. The experimental results showed that after 1,200 cycles, the CNT capacitances of M-120//GO, M-160//GO, and M-180//GO reached 43.3%, 87.3%, and 46.5% of the initial specific capacitance, respectively, indicating that the electrochemical storage performance of M-160//GO capacitor is good and the stability is high. The application in sports equipment can improve the battery capacity and enhance the practicability of the equipment.

## 1 Introduction

Sports equipment is the material basis necessary for sports activities. The quality of sports equipment is an important factor affecting the quality of sports competitions. In competitive sports, the advancement of sports equipment has a great impact on sports performance. Sports equipment is an essential part of sports activities. It can ensure the safety of athletes, improve the performance of the game, and ensure the fairness of the game. The purpose of the development of sports equipment has always been to ensure the fairness of the competition. It can not only improve the athletic level of athletes, but also reduce the risk of injury to athletes. Conjugated nano-materials are a kind of new materials that have attracted much attention in the field of materials science. They have unique structures and properties, including carbon nanotubes, graphene, nano-metal particles and so on. These materials are highly regarded for their high strength, light weight, electrical conductivity, thermal conductivity and chemical stability. It has application potential in many fields, such as electronics, medical equipment, energy storage and transmission. However, although these materials have made remarkable achievements in other fields, their application in the field of sports equipment has not been fully explored. Traditional sports equipment usually uses traditional materials, such as metal, plastic and rubber. Although these materials have been widely used in sports equipment, there are some limitations. Conjugated nano-materials have the potential to improve sports equipment by providing new performance characteristics, so as to improve the performance of athletes, enhance the durability of equipment and provide better user experience. The scope of sports science research has expanded, and new disciplines have emerged from the mutual penetration and development of sports and other disciplines. In such a large environment, combining nanotechnology, an emerging technology with the sports industry, is in line with the needs of the current development trend of sports science. With the development of sports, people’s dependence on science and technology is increasing, and nanomaterial technology is an emerging field of science and technology, and its composite material technology has broad application prospects in the sports industry. Therefore, this paper aims to explore the use of composite technology in nanotechnology for the research of sports equipment, and analyze the interaction between the two through the association rule method. The preparation process and experimental formula of nanomaterials are studied, the correlation between carbon nanotubes and the performance of sports equipment is analyzed, and the organic combination of materials and sports equipment is of great significance to the development of sports equipment industry and the improvement of sports level.

The innovation of this paper is that by discussing the application of nanotechnology in sports equipment, it can achieve theoretical integration in sports equipment. Thereby, its application in sports equipment is more extensive, so as to achieve better comprehensive performance, thereby improving the practicability of sports equipment. Specifically in the field of sports equipment design, conjugated nanomaterials represented by carbon nanotubes and graphene not only have excellent electrical conductivity, thermal conductivity and mechanical properties, but also perform well in electrochemical energy storage performance, which makes sports equipment An important step has been taken in lightweighting, increasing strength and improving battery performance. By optimizing the design of sports shoes, equipment and equipment, conjugated nanomaterials provide unique solutions to improve sports equipment performance, enable more advanced functional designs, and create innovative sports experiences of the future.

## 2 Related work

Advanced material resources are an important factor to improve the scientific and technological level of a country’s sports equipment. At present, many countries in the world, especially developed countries, are vigorously developing and using various high-tech and new materials to promote the development of sports equipment. For example, Shalaby (2020) presented the current and future global research progress on new nanomaterial wearable strain sensors, and discussed their role in the sports equipment industry in detail. Kimura et al. (2017) believed that single-leg squatting with exercise machines that produce different resistances can increase glute medius load and prevent knee valgus torque. Li et al. (2021) mainly expounded the application of titanium alloys in sports equipment, and carried out an optimized design. It can be seen that the emergence of these new materials has changed the development of sports equipment to varying degrees, but most of the above scholars have stayed in theoretical research and lacked scientific algorithms for practical operation.

Conjugated nanomaterials are a kind of nanomaterials with conjugated structure, and their electronic structures show a series of conjugated bonds or π -electron systems. This structural feature makes these nano-materials have unique properties in electronics, optics, magnetism, electrical conductivity and thermal conductivity, which has aroused extensive research interest. The association principle in data mining is also commonly used by many researchers in the field of sports, and has achieved certain research results. For example, Wang et al. (2017) studied a main damage model of braided composites obtained by data mining technology, which has good structural stability and good damage resistance, and is a very ideal sports protection material. In order to overcome the fuzziness of the industrial design of human adaptive motion equipment, Ruan and Li (2020) used fuzzy mathematical theory as an analysis tool. According to its actual characteristics, the essence of industrial design under the fuzzy theory was emphatically discussed. Liu and Ma (2020) took patients with hyperlipidemia as experimental subjects, and used high-intensity exercise equipment made of nanomaterials for exercise rehabilitation training to explore the correlation between nanomaterials and patients with hyperlipidemia. Tong-Zhigang (2021) combined association rules with Support Vector Machines (SVM), used association rules to extract features of sports training equipment, and determined relevant rules. The research of these scholars has promoted the expansion of sports equipment materials to a certain extent, but the scientific algorithms they used are not perfect and need to be further optimized.

## 3 Application of conjugated nano-materials in sports equipment

### 3.1 Application of graphene technology

Conjugated nanomaterials, It has excellent electrical conductivity, light weight and high strength, excellent thermal conductivity and multi-functionality. Especially graphene technology, have great potential in the field of sports equipment, which can change the design and performance of sports equipment. Conjugated nano-materials provide new possibilities for the design and manufacture of sports equipment because of their unique properties and potential application fields. By using these materials, we can improve the performance of equipment, reduce the burden on athletes, and promote innovation in the field of sports equipment. Through the related processing technology, the best performance of conjugated nano-materials in practical application can be realized.

Conjugated materials are widely used in the field of sports equipment. The most typical application is in reinforced parts of sports shoes and sports equipment. Carbon nanotubes and graphene are compounded with natural rubber and used in the outsoles or soles of sports shoes., can improve the wear resistance, electrical conductivity and thermal conductivity of the sole, thereby enhancing the performance and comfort of the shoe. In addition, when manufacturing reinforced parts of tennis rackets, embedding carbon nanotubes and graphene into elastic materials can improve the strength, stiffness and durability of these parts, while reducing the overall weight, greatly improving the performance of sports equipment.

Conjugated materials have strong market prospects and commercialization potential. The lightweight design of conjugated nanomaterials has significant potential in sports equipment. By reducing the weight of equipment, athletes can obtain a more flexible and efficient sports experience, and share the same benefits. The excellent electrical and thermal conductivity of the yoke nanomaterials is expected to improve the overall performance of the equipment, providing better support and comfort. Secondly, the electrochemical properties of conjugated nanomaterials make them ideal for battery technology. In sports equipment, this may manifest as longer-lasting electric assistance, longer battery life in smart sports equipment, and higher performance in sports monitoring equipment., which shows that its commercialization potential is huge. The most important thing is environmental protection. In response to the national environmental protection call, the use of conjugated nanomaterials is more environmentally friendly. This is consistent with today’s focus on sustainable development and environmental protection, and may bring opportunities for environmentally friendly images to the brand.

Graphene is a single-layer two-dimensional lattice structure composed of carbon atoms, which has excellent electrical conductivity, thermal conductivity and mechanical strength, and is one of the most famous conjugated nanomaterials. Graphene has a wide application potential, including electronic devices, sensors, supercapacitors, flexible display screens, protective materials and so on. Graphene can be applied to sports equipment.

Graphene is widely used in sports equipment, especially the impact of conductivity on sports equipment. In sportswear, graphene conductive fibers are used to make smart textiles with breathability and heat preservation properties. In smart sports parts, the conductivity of graphene is used to make smart gloves, headscarves, etc., for monitoring physiological indicators and transmitting data. Or provide heating function.

In recent years, due to the government’s policy orientation and people’s emphasis on physical health, mass sports has developed rapidly. Sports activities are inseparable from sports equipment, and the development of sports has also promoted the development of sports equipment (Barbosa, 2018). Public sports have a wide range of participation and a large number of participants, so there is a wide range of demand and use of public sports equipment. With the rise of sports and leisure, mass sports has become an important part of China’s sports industry (Ge, 2017).

**FIGURE 1:**
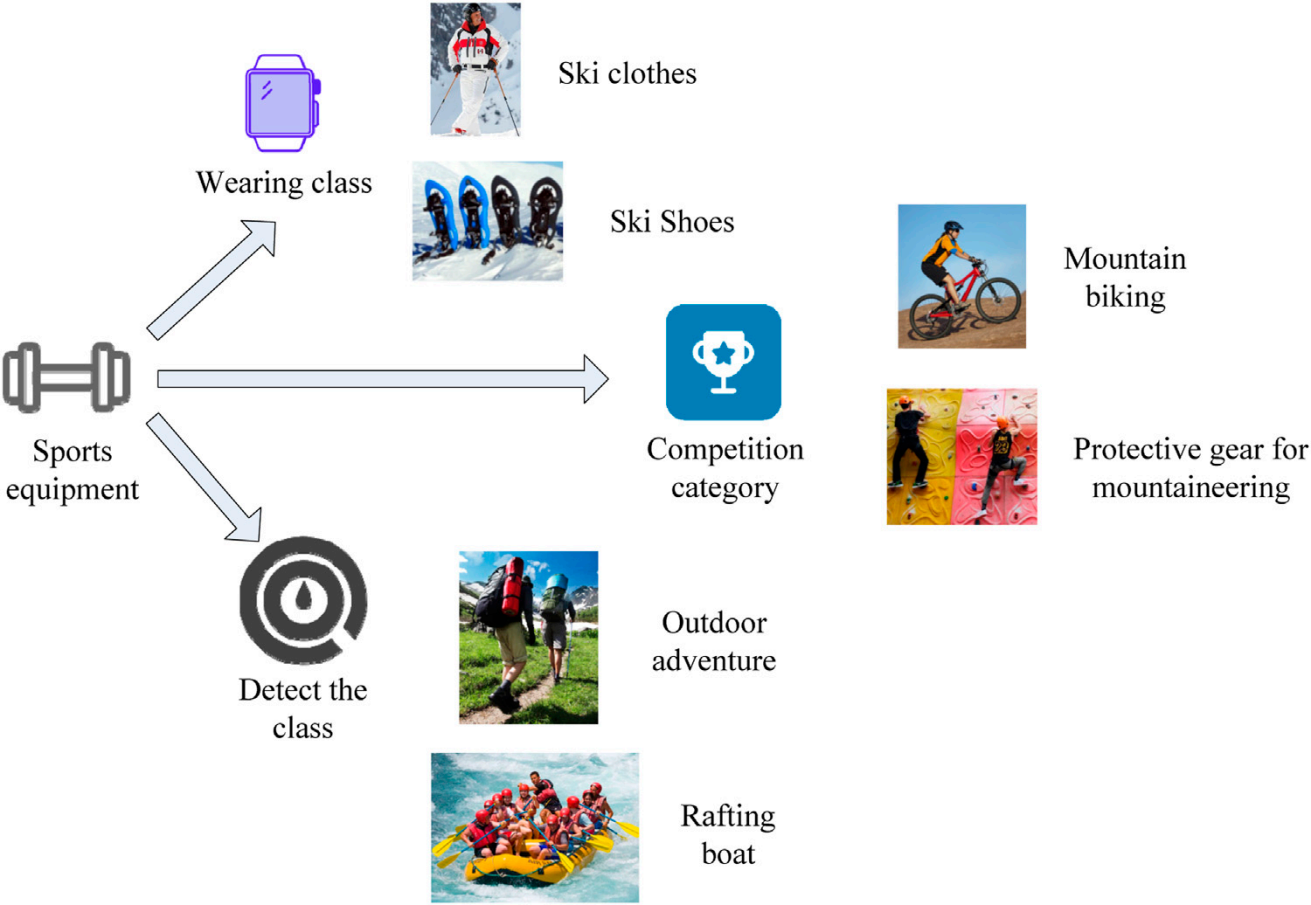
Typical projects where graphene technology can be applied in mass sports.

From the perspective of various application methods in Figure 1, in addition to common sports such as badminton, basketball, and tennis, there are also extremely challenging sports such as ice and snow, rock climbing, and outdoor exploration. These are excellent areas to utilize graphene technology (Grande et al., 2018). Graphene sports equipment can be widely used in various sports equipment, from large to small, and from land to water. With the wider application of graphene technology in sports equipment, graphene technology would no longer be a scarce technology in sports equipment; with the widespread use of graphene sports equipment in the field of mass sports, it would be quickly accepted by people.

At present, the preparation technology of graphene rubber composites has been quite mature, including three categories of latex blending, solution blending and mechanical blending (Graziosi et al., 2017; Koury, 2018). This article uses mechanical blending method to prepare graphene rubber composite materials, uses a universal material testing machine to measure the strength, hardness, elastic modulus, etc. of the material, uses thermogravimetric analysis to measure the stability of the material, and uses electrochemical testing. Conductivity, etc.

**FIGURE 2:**
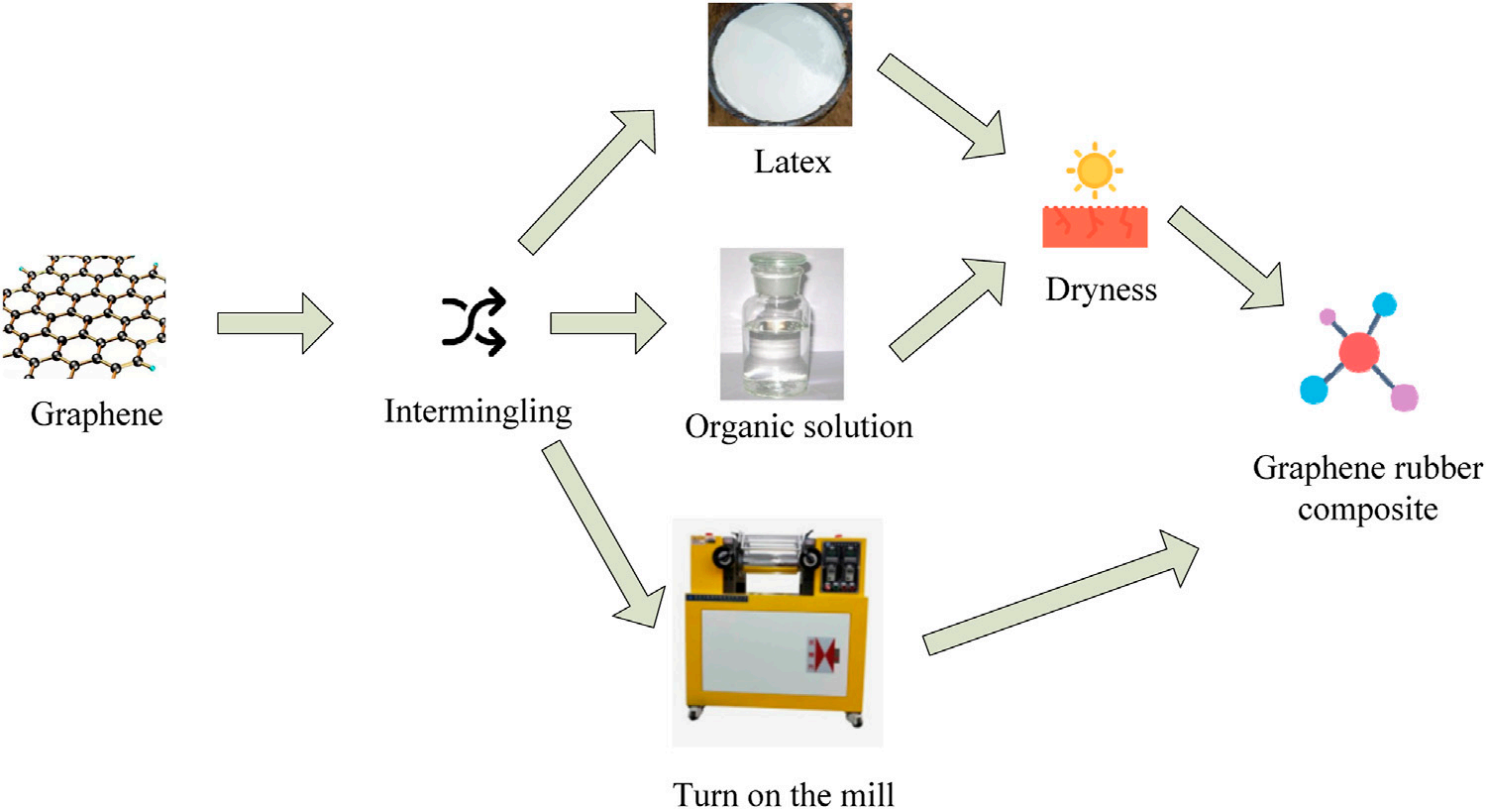
Fabrication method of graphene composites.

Figure 2 is the manufacturing method steps of three kinds of graphene composite materials. The first mixing method is to mix graphene and latex to obtain a composite material after drying. This method does not use organic solvents and can reduce environmental pollution. The second method is the organic solution mixing method. Graphene is dissolved in an organic solution and then dried to obtain a uniformly dispersed composite. This method can achieve optimal polymerization of mixtures and nanomaterials, but this method is complicated and difficult to operate in the solvent extraction process. The third method is the mechanical mixing method. The composite material is directly obtained by mixing various ingredients using an open mill. This method is simple and convenient, and is the best method for industrial mass production (Barbosa, 2018; Yunus et al., 2019).

The specific preparation steps of graphene rubber composite materials are as follows. First, prepare a GO aqueous solution at a concentration of 1 mg/mL, disperse it ultrasonically for 25 min, and add caprolactam (CPL) and graphene oxide (GO) at a mass ratio of 1:3. Stir in CPL and continue ultrasonic dispersion for 4 h to obtain CPL-GO. Then stir the CPL-GO aqueous solution and NR emulsion at a certain mass ratio at room temperature for 40 min, and use 8% mass fraction of calcium chloride solution to flocculate NR. The latex is dried in a drying oven at 50°C until the mass is constant. CPL-GO, zinc oxide, stearic acid, accelerator D, accelerator DM and sublimated sulfur are mixed in an open mill, and finally vulcanized on a vulcanizer, the vulcanization temperature is 150°C, the vulcanization pressure is 10 MPa, and the vulcanization time adopts the positive vulcanization time (t90), that is, the graphene rubber composite material CPL-GO/NR is obtained.

 Nowadays, graphene rubber is widely used in the production of automobile tires. Due to the uneven distribution of graphene in automobile tires, it is difficult to be made into graphene rubber (Caporaso et al., 2018). The process adopts a variable gap mixing process. By setting shear steps on the long and short sides, the continuous cycle of shear separation and dispersion steps is realized, and the optimal temperature of shearing, dispersing, pouring, and reconciling is achieved. This ensures that the graphene, which is difficult to be uniformly dispersed, is uniformly dispersed in the rubber (Meier et al., 2019). It has laid a good foundation for the popularization and application of graphene rubber in the future. At present, graphene rubber has a variety of production technologies and has good application prospects.

### 3.2 Association mining algorithm

In the quality evaluation of sports equipment, the statistical data of the evaluation indicators are usually very limited, and the data have great fluctuations, and the distribution law is not clear. This method has the characteristics of less original data, simple principle, and easy mining of data rules, especially using the grey relational analysis method to deal with such problems effectively. Its essence is to analyze and compare the geometrical difference between each factor curve and the obtained curve (Stuart et al., 2020). Using the grey relational analysis method, from the perspective of influencing factors, the main characteristics of influencing factors and the differences of influencing factors are studied. Assuming that there are m samples of sports equipment waiting for evaluation, the evaluation matrix formed by n evaluation indicators is expressed as:

$$Y={\left[y_{ij}\right]}_{m\times n}$$ (1)

$y_{\text{ij}}$ represents the observation data of the *j*th sports equipment quality evaluation index of $Y_{i}$ elements, and then the steps of grey relational analysis and calculation are as follows:

The sequence of surface features is represented as:

$$Y_{0}=\left(y_{01},y_{02},...,y_{0n}\right)$$ (2)

The aligned sequence of system elements to be analyzed is:

$$Y_{i}=\left(x_{i1},x_{i2},...,x_{in}\right),\left(i=1,2,...,m\right)$$ (3)

Then the difference sequence value is calculated:

$$\Delta \left(j\right)=\left|y_{0j}-y_{ij}\right|$$ (4)

Obtaining the range value is divided into two levels of maximum range and minimum range. Q and q represent the maximum and minimum range respectively:

$$\left\{\begin{array}{l} Q=\max_{i}\max_{j}\Delta \left(j\right)\\ q=\underset{i}{mix}\underset{j}{mix}\Delta \left(j\right) \end{array}\right\}$$ (5)

The formula for calculating the correlation coefficient is:

$$\gamma \left(y_{0j},y_{ij}\right)=\frac{q+\rho Q}{\Delta \left(j\right)+\rho Q}$$ (6)

Among them, ρ represents the discrimination coefficient, and the correlation degree is expressed as:

$$\gamma_{0i}=\frac{1}{n}\sum_{j=1}^{n}\gamma \left(y_{0j},y_{ij}\right)$$ (7)

Then, the information entropy is calculated for each index of sports equipment, and the weight of each index is obtained (Liu et al., 2021). The size of the weighting coefficient indicates the degree of influence of the index on the entire system, and the weighting coefficient calculated by the entropy method can more intuitively and effectively reflect the relationship between the indexes.

After normalizing the evaluation matrix Y, it is:

$$Y={\left[y_{ij}\right]}_{m\times n},y_{ij}$$ (8)

At this time, the formula for calculating the entropy value $F_{j}$ of each evaluation index is:

$$F_{j}=\frac{\sum_{i=1}^{m}R_{ij}\ln R_{ij}}{\ln m}$$ (9)

Among them, $R_{ij}$ represents the standardized index:

$$R_{ij}=\frac{y_{ij}}{\sum_{i=1}^{n}y_{ij}}$$ (10)

Then the weight $\upsilon_{j}$ of the *j*th indicator is:

$$\upsilon_{j}=\frac{H_{j}}{\sum_{j=1}^{n}H_{j}}$$ (11)

$$H_{j}=1-F_{j}$$ (12)

In the above formula, $H_{j}$ is the difference coefficient of the *j*th sports equipment evaluation index.

In the decision-making of multiple indicators, there are often many decision indicators, and the weight difference of each indicator is very small. Therefore, the multiplicative synthesis method is usually used to weight each indicator in combination to maximize the importance of each indicator (Zhu, 2018). The specific weighting formula is:

$$\omega_{j}=\frac{\varepsilon_{j}\upsilon_{j}}{\sum_{j=i}^{n}\varepsilon_{j}\upsilon_{j}}$$ (13)

Among them, $\omega_{j}$ represents the objective weight of the *j*th evaluation index, and $\varepsilon_{j}$ represents the weight of the *j*th index after the weighting method.

After calculating the weight matrix A, assuming that the correlation matrix is B, the gray correlation matrix C is expressed as:

$$C=A\times B^{Y}$$ (14)

The modeling of the sports equipment and CNT studied in this paper conforms to various technical performance indicators, so the technical performance indicators of the components are not considered in the modeling. At the same time, only considering the relationship between mechanical properties and capacitance, the $I_{0}$ model of CNT capacitance can be obtained as follows:

$$I_{0}=\sum_{i=1}^{D}\sum_{j=1}^{m}a_{ij}x_{ij}$$ (15)

$$\sum_{i=1}^{m}a_{ij}=1,j=1,2,...,D$$ (16)

Among them, $a_{ij}\in \left\{0,1\right\}$ represents the loss of the *j*th type under the *i*th type of material, and $x_{ij}$ is the electricity consumption ratio. D is the number of materials; m is the number of materials to be tested.

Then the final selection is:

$$C=H\cdot I_{0}+\left(1-H\right)\cdot L$$ (17)

In the formula, L is the average performance of the sports equipment; H is the weight coefficient of the sports equipment formula.

### 3.3 Correlation of main raw materials of sports equipment

Based on the idea of correlation algorithm, this paper links the main materials of sports equipment with nanomaterials, in order to formulate different strategies for sports equipment according to the characteristics of different quality factors of sports equipment.

As can be seen from Table 1, among the four quality elements of sports equipment, the parts are in the key control area, so special attention should be paid to them during assembly; the quality element in production belongs to the quality-oriented area, with high quality sensitivity, and the quality of the product can be improved by strengthening quality control and appropriate expenses; the material is in the optimal area of the strategy, which can minimize the cost while maintaining a low quality sensitivity; since the method is in the quality reserve area, its cost sensitivity and quality sensitivity are relatively low. Therefore, as long as the original method is maintained, the failure or accident caused in the operation of the equipment can be reduced.

**TABLE 1:** Coordinate values of grey relational matrix of sports equipment.

Quality factor	Cost (%)	Quality
Materials	15.38	0.3972
Components	64.98	0.579
Manufacturing	10.11	0.9497
Craft	9.53	0.4439

## 4 Experiment of CNT/NR composites

### 4.1 Performance of CNT composites by nanofabrication

The application of conjugated nano-materials in sports equipment has great potential, which can improve the performance, lightweight, durability and sustainability of the equipment. Nano-materials such as graphene and carbon nanotubes have excellent strength and lightweight characteristics, and can be used to manufacture lighter and stronger equipment, such as bicycle frames, snowboards, golf clubs and tennis rackets.

In this paper, Carbon Nanotube/Natural Rubber (CNT/NR) composites were studied by HAAKE torque rheometer. The rotor speed, initial temperature, stirring time and other factors of the HAAKE mixture were studied to study the physical and mechanical properties, dynamic mechanical properties of CNT/NR and the dispersion properties of carbon nanotubes, and the optimal mixing process parameters were obtained. An initial temperature of 60°C and a mixing time of 10 min were set; the effect of the rotor speed on the physical and mechanical properties of CNT/NR composites was investigated at 40, 50, 60, 70, and 80 r/min.

It can be seen from Table 2 that it has the highest tensile strength at the rotor rotation speed of 80 r/min, which is due to the better distribution of CNTs in NR. While at the speed of 40 r/min, the tensile strength of 100% and 200% stress are the largest among all the speeds. When the rotation speed is 60 r/min, it has the maximum fracture energy, reaching 23.99J. As for the material hardness, there is a slight fluctuation with the change of rotation speed, and the hardness reaches the maximum at 50 r/min.

**TABLE 2:** Physical and mechanical properties data of CNT/NR composites at different rotational speeds.

Rotating speed	Modulus at 100%/MPa	Modulus at 200%/MPa	Tensile strength/MPa	Elongation at break/%	Tear strength/ $KM\cdot m^{-1}$	Fracture energy (J)	Hardness (MPa)
40 r/min	2.43	6.64	26.01	633	46.05	22.40	32.20
50 r/min	2.31	6.26	27.26	655	44.71	23.91	34.61
60 r/min	2.25	6.21	26.34	657	46.20	23.99	33.04
70 r/min	2.22	6.00	26.41	656	47.81	22.07	34.17
80 r/min	2.13	5.83	27.36	682	45.95	21.62	31.29

**FIGURE 3:**
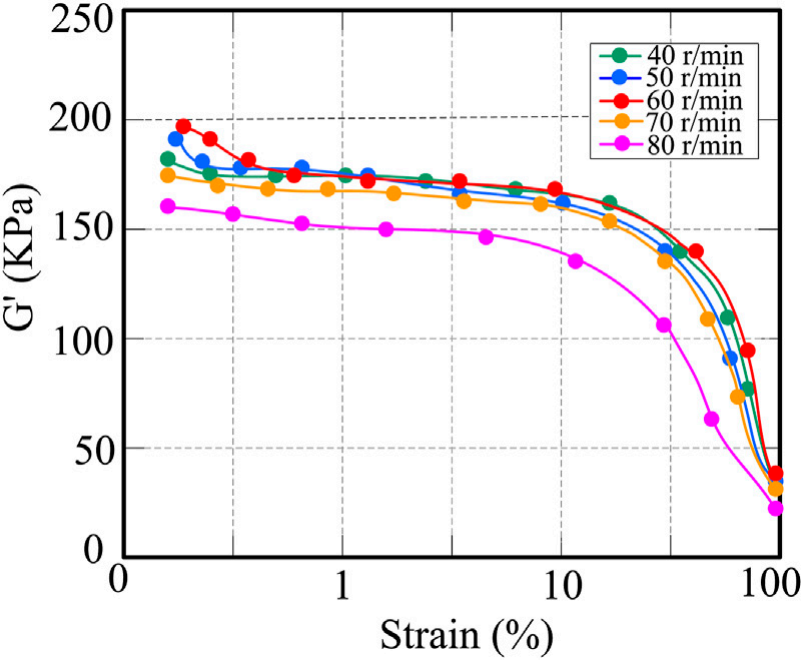
Variation of storage modulus of CNT/NR composites at different rotational speeds.

Figure 3 shows the relationship between energy and strain of CNT/NR composites. When the strain amplitude is low, except when the rotational speed is 80 r/min, the other rotor rotational speeds have little effect on the energy modulus of CNT/NR. As the strain increases, the storage modulus decreases, while at smaller strains, the energy modulus remains basically unchanged. The effects of the initial temperature on the mechanical properties and CNT dispersion characteristics of CNT/NR were then analyzed. The initial temperatures were set at 40°C, 50°C, 60°C, 70°C, and 80°C, respectively.

Table 3 shows the properties of the CNT/NR composites at the initial temperature. The data results show that when the initial temperature is 50°C, the tensile stress is 100%, the tensile stress is 300%, and the tensile strength is the maximum. The higher the mixing temperature, the greater the penetration and powder absorption of the rubber particles, and the more unfavorable the dispersion of the rubber. At the same time, in the mixing process, there are still problems such as scorching and over-smelting, which lead to the deterioration of the quality of the rubber compound. Compared with other temperatures, at the initial temperature of 50°C, the mechanical properties of the composite material are superior. This is mainly because at this temperature, the plastic movement and powder eating of rubber molecules is also conducive to the dispersion of fillers and other additives, thereby improving the quality and mechanical properties of the mixture.

**TABLE 3:** Data on physical and mechanical properties of CNT/NR composites at different temperatures.

Temperature (°C)	Modulus at 100%/MPa	Modulus at 300%/MPa	Tensile strength/MPa	Elongation at break/%	Tear strength/ $KM\cdot m^{-1}$	Fracture energy (J)	Hardness (MPa)
40	2.1	5.92	25.32	654	46.5	20.65	30.15
50	2.4	6.14	27.26	656	45.7	21.33	31.24
60	2.3	5.92	26.34	667	46.1	22.01	30.03
70	2.3	6.13	25.41	655	41.1	20.30	29.38
80	2.3	5.91	26.36	681	44.6	18.71	29.27

**FIGURE 4:**
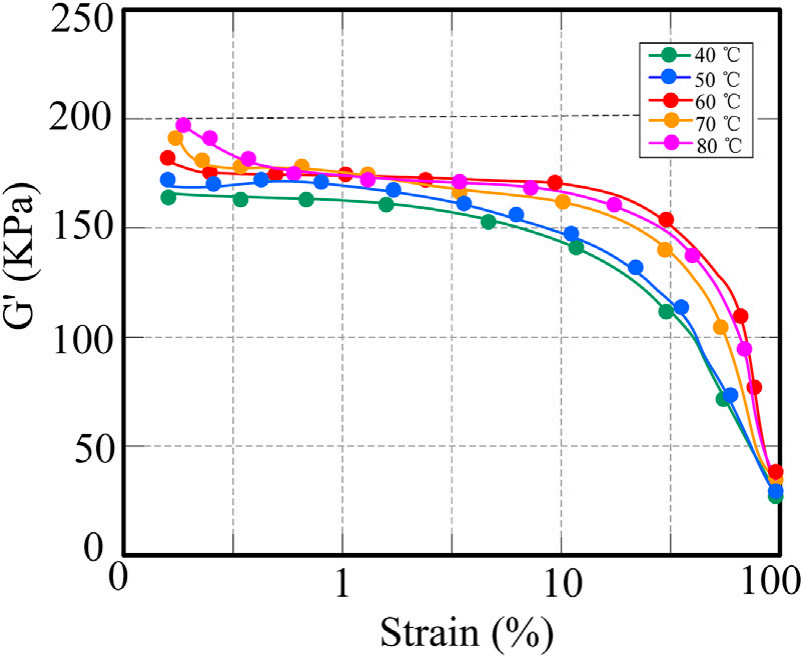
Variation of storage modulus of CNT/NR composites at different temperatures.

In order to analyze the effect of starting temperature on the change of storage modulus of CNT/NR composites, the composites were analyzed by NR processing analyzer, and the change curve as shown in Figure 4 was drawn.

The results in Figure 4 show that at lower strains, the elastic modulus has little relationship with the strain. When the strain increases, the energy modulus decreases. At 40°C and 50°C, the storage modulus is relatively small. When the strain increases, the network structure of the filler would be destroyed, and the rubber of the filler coating would be released, resulting in a decrease in the effective filler amount of the filler and a sharp drop in the storage modulus. However, compared with the initial temperature of 40°C, the mechanical properties at 50°C are better, and the energy consumption is also less. In general, it is ideal to set an initial temperature of 50°C.

Finally, the effects of mixing time on the physical and mechanical properties and dynamic mechanical properties of CNT/NR composites were analyzed. The mixing time was set to 8 min, 10 min and 12 min, respectively.

It can be seen from the data in Table 4 that at 10 min and 12 min, the 100% and 300% tensile stress and tensile strength after mixing are higher than the mixing time of 8 min. When the mixing time was 12 min, the tensile strength, elongation rate and other performance indicators of the CNT/NR composite were not significantly different from those of 10 min, and the mechanical properties of the materials were all better. This is because the dispersibility of CNTs is better in this case. After the mixing time of 8 min, the performance of the mixture decreased because the mixture did not reach the optimal dispersion state. In the case of obtaining high-quality rubber compounds, reasonable mixing time can improve production efficiency and save energy. To improve product performance and yield, a mixing time of 10 min was chosen. Based on the above analysis results, the optimal process conditions for CNT mixing were concluded as follows: rotor speed of 40 r/min, initial temperature of 50°C, and mixing for 10 min.

**TABLE 4:** Comparison of physical and mechanical properties of CNT/NR at various mixing times.

Rotating speed (min)	Modulus at 100%/MPa	Modulus at 300%/MPa	Tensile strength/MPa	Elongation at break/%	Tear strength/ $KM\cdot m^{-1}$
8	2.2	5.71	26.42	678	45.7
10	2.4	6.35	27.26	655	44.8
12	2.4	6.38	28.34	696	45.8

**FIGURE 5:**
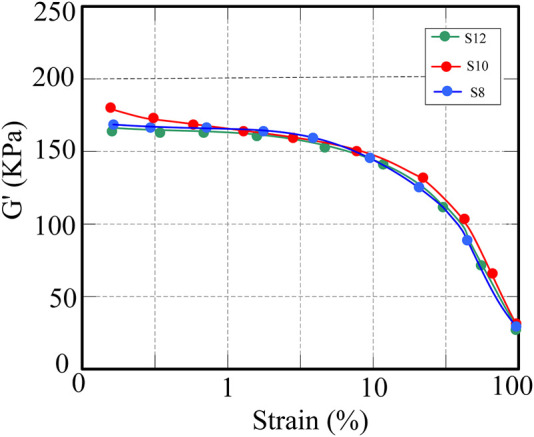
Trend of storage modulus of CNT/NR composites at different mixing times.

It can be seen from the curve in Figure 5 that the energy modulus of the composite material hardly changes when the strain value is smaller. However, as the strain increases, the storage modulus also decreases. The reason for this may be similar to the dispersibility of CNTs. This is mainly due to the fact that the composites did not reach the optimal dispersion effect within 8 min.

**FIGURE 6:**
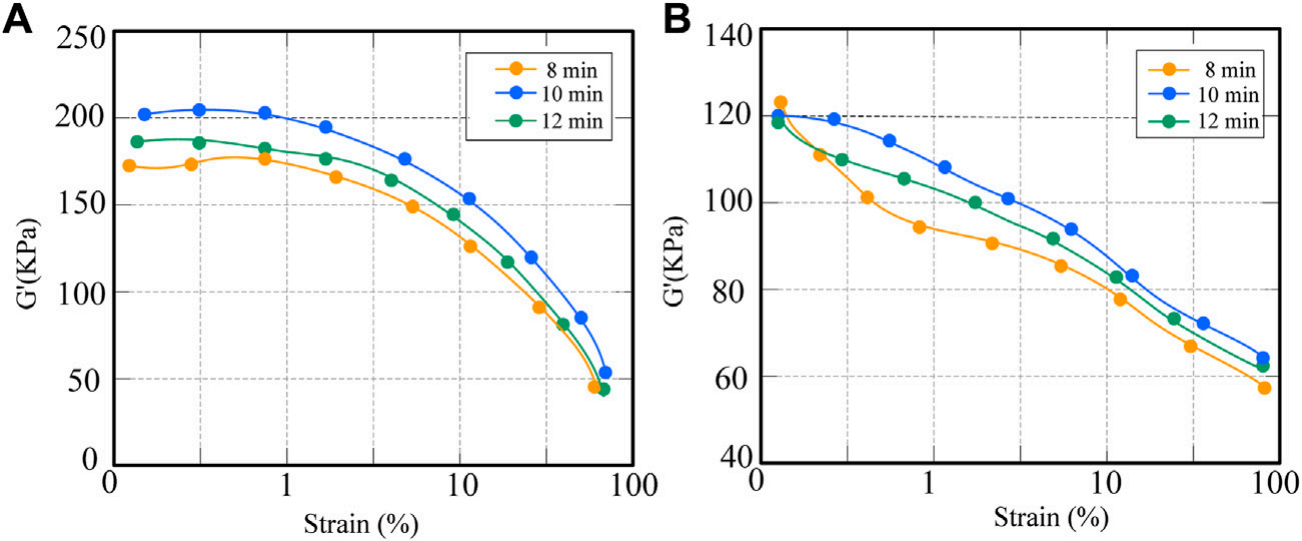
Changes in dynamic mechanical properties of CNT/NR hybrids under different mixing times. **(A)** Effect of storage modulus. **(B)** Effect of loss factor.

It can be seen from Figure 6A that after mixing for 10 min, the G′ of CNT/NR is the largest at this time, and the G′ of CNT/NR with a mixing time of 8 min is the smallest. It can be seen from Figure 6B that when mixing for 10 min and 12 min, the G′ is smaller when the mixing time is 12 min. The results show that the CNT/NR composite has good physical mechanical and kinetic properties at low temperature and low speed, and has good dispersibility.

### 4.2 Graphene galvanostatic charge-discharge test

This test also used asymmetric supercapacitors to examine the electrochemical storage properties of graphene electrode materials. The supercapacitor is composed of positive and negative electrodes, the number of cycles is 1,200 times, the separator is NKK-MPF30AC-100 type water-based separator, and GO is the negative electrode. According to the different cathode materials, they are divided into M-120/GO, M-160/GO, and M-180/GO.

**FIGURE 7:**
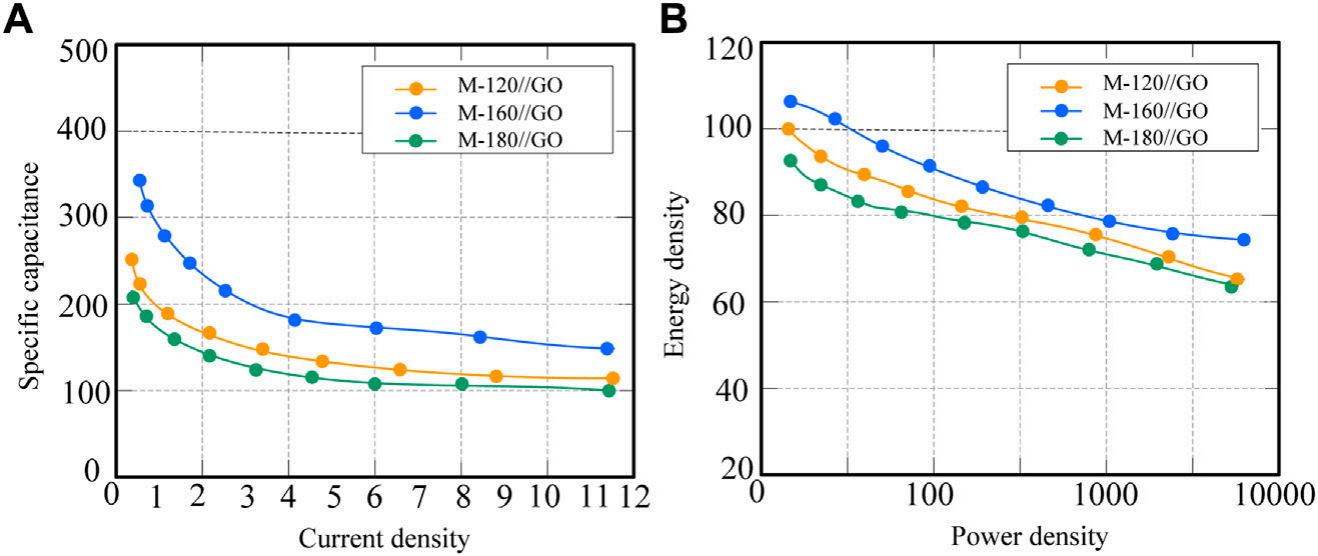
Individual data plots for different supercapacitors at different current densities. **(A)** Specific capacitance diagram **(B)** Energy density and power density diagrams.

From the data in Figure 7A, it can be seen that M-160/GO has the largest discharge time, followed by M-120//GO, and M-180/GO has the shortest discharge time. Figure 7B is a more intuitive comparison of the storage properties of the three electrode materials. The comparison results show that the M-160/GO supercapacitor has good electrochemical superiority.

By analyzing the discharge curve of the M-160/GO supercapacitor, the specific capacitance, power density and energy density under different current densities were obtained. The specific data are listed in Table 5.

**TABLE 5:** Specific capacitance, energy density and power density of M-160//GO at different current densities.

$I\left(Ag^{-1}\right)$	$C\left(Fg^{-1}\right)$	$E\left(Whkg^{-1}\right)$	$P\left(Wkg^{-1}\right)$
0.2	343.07	123.00	161.03
0.4	286.52	110.56	253.89
0.6	264.53	78.43	502.60
0.8	250.21	81.23	612.02
1	240.62	85.57	800.30
2	201.53	71.68	1,600.82
3	184.80	65.68	2,300.00

It can be seen from Table 5 that when the current density is 0.2 A $g^{-1}$ , the specific capacitance can reach 343.07 F $g^{-1}$ , and the energy density and power density are 123.00 ${\text{Whkg}}^{-1}$ and 161.03 ${\text{Wkg}}^{-1}$ , respectively. As the current density increases, the specific capacitance and energy density decrease accordingly, while the power density increases accordingly.

### 4.3 Cyclic stability test of graphene

**FIGURE 8:**
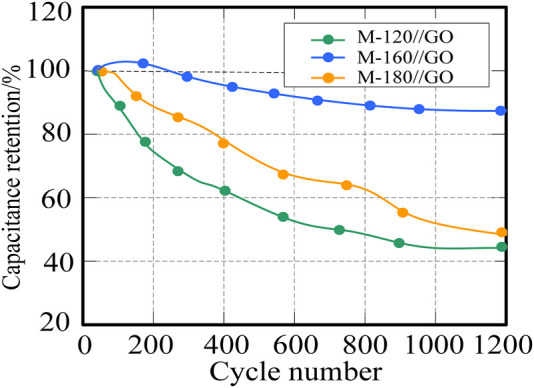
Cycling stability test of different supercapacitors.

Figure 8 is a graph of the percentage specific capacitance loss of graphene capacitors of M-120/GO, M-160/GO/GO and M-180/GO at 8 A $g^{-1}$ current density. The results show that the specific capacitance of the three graphene capacitors decreases with the increase of cycle time. After 1,200 cycles, the capacitance of the M-160/GO capacitor decreases the most, and finally decreases to 43.3%. However, the capacitance of M-160//GO capacitors has the smallest decrease in capacitance, and the capacitance of M-160/GO can still maintain 87.3%, while the capacitance of M-180//GO finally drops to 46.5%. It shows that M-160//GO capacitor has better electrochemical storage performance and higher stability among the three capacitors.

### 4.4 Wear resistance of composite materials and adaptability in different environments

In order to further explore the performance of the composite materials in this article, the wear resistance and corrosion resistance in humid environments of the conjugated nanomaterials in this article and the traditional materials in the original article are compared. The details are shown in Table 6. The corrosion resistance is determined by combining four Soak different materials in rain water at the same time, and check the effect after half a month. From the perspective of wear rate, conjugated nanomaterials have reached 0.02 mm³/N, which is quite good. Compared with wooden materials, it has dropped by 0.13 mm³/N. The best wear rate is that of metal materials, which is only 0.001. For quality loss Percentage, the conjugated nanomaterials in this article are only 1.4%, and the metal materials have the largest mass loss, reaching 8.2%. Overall, the conjugated nanocomposites in this article achieve good performance in wear resistance and corrosion resistance.

**TABLE 6:** Wear resistance and corrosion resistance of different materials.

Material	Wear rate (mm³/N)	Quality loss percentage (%)
Conjugated nanomaterials	0.02	1.4
Wooden material	0.15	5.9
Rubber material	0.79	6.5
Metallic material	0.001	8.2

### 4.5 Comparison of cost-effectiveness and service life of different materials

**FIGURE 9:**
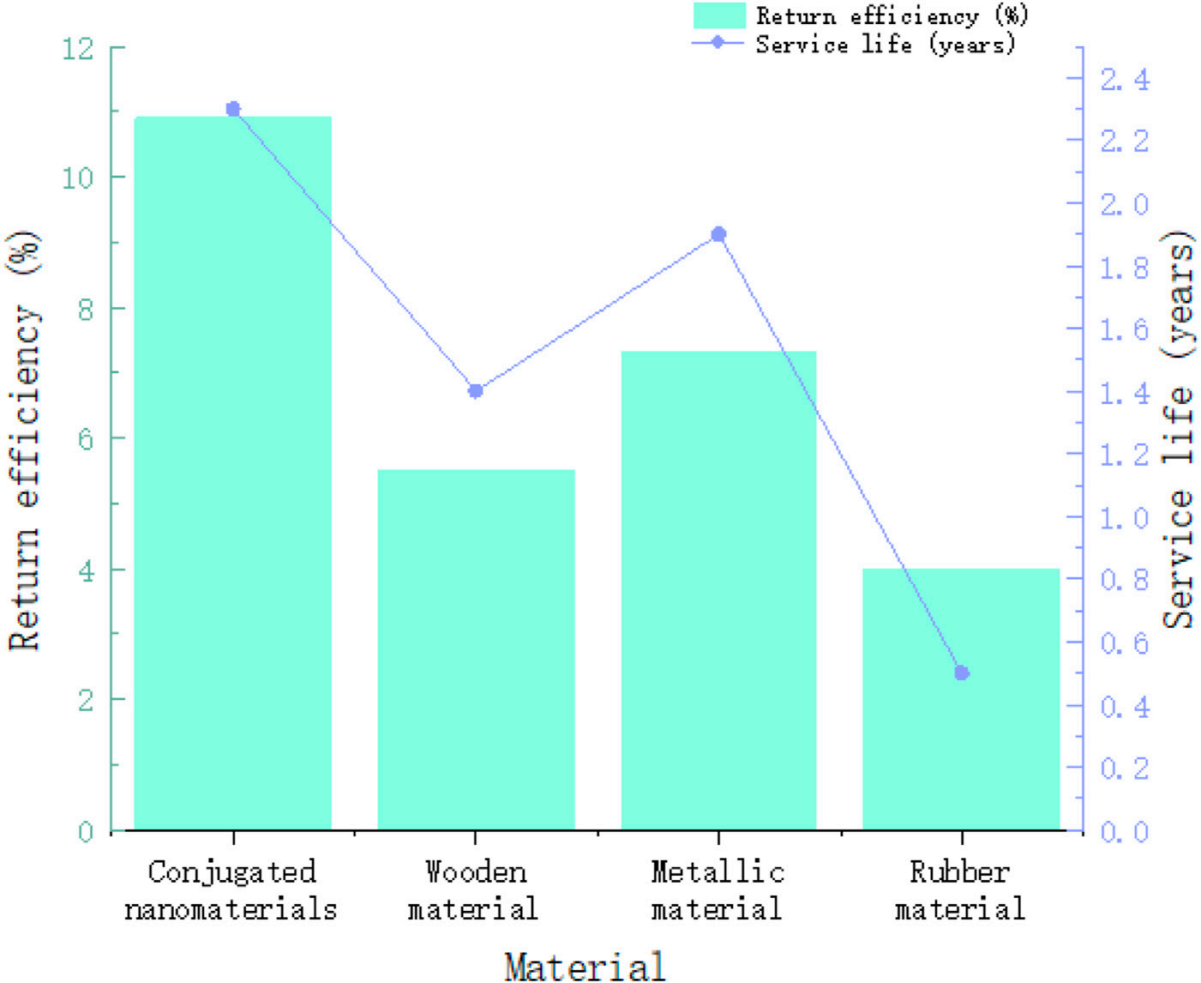
Comparison of cost-effectiveness and service life of different materials.

In order to explore the cost-effectiveness of materials, the return benefit is now measured by the ratio of revenue to cost. The higher the ratio, the better the return benefit and the relatively lower cost. As can be seen in Figure 9, the return benefit of conjugated nanomaterials has reached 10.9%. It can be seen that the proportion of cost compared to revenue is very small, while the revenue of rubber materials accounts for a lower proportion of cost compared to cost, only 4.0%. The benefits are very small and the costs account for much more. Regarding the service life, the adaptive life of conjugated nanomaterials has reached 2.3 years, which is 0.4 years longer than that of metal materials. Rubber materials are the most susceptible to wear and tear, and their service life is only half a year. Overall, the conjugated nanomaterials in this article show good performance.

### 4.6 Satisfaction feedback scores for sports equipment made of different materials

**FIGURE 10:**
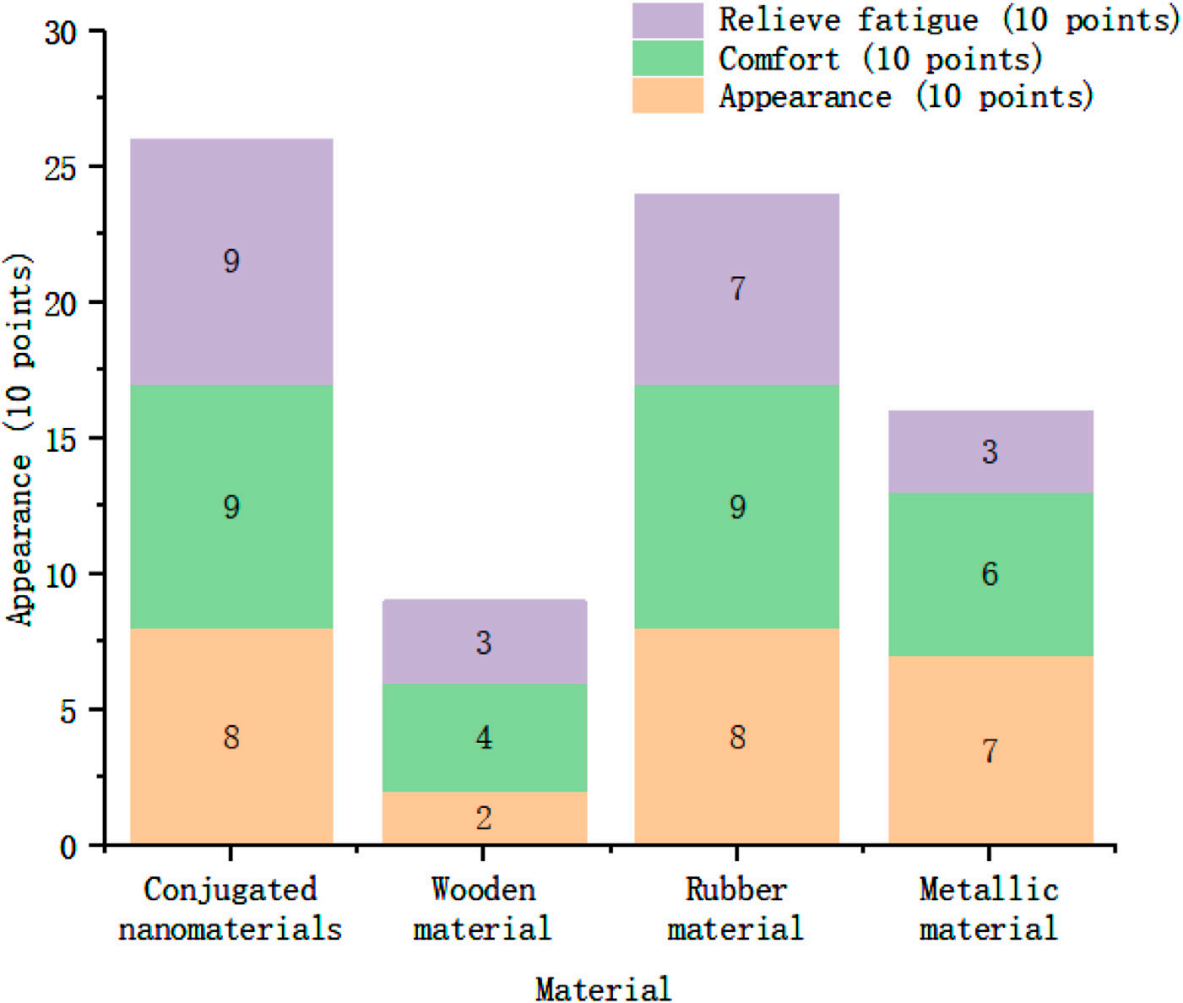
Satisfaction feedback scores for sports equipment made of different materials.

In order to explore the effect of using sports equipment, an on-site questionnaire survey was conducted with 500 samples. The evaluation indicators were the appearance, comfort and fatigue relief of tennis rackets. The specific results are shown in Figure 10. Overall, the stacked histogram of conjugated nanomaterials has the highest score, reaching 26 points, accounting for 86.7% of the total score. The lowest score is the racket made of wooden materials, with a score of only 9 points. It can be seen that users are more inclined to conjugated nanomaterials, but the appearance and suggestions made by users need to continue to be improved.

## 5 Conclusion

In this paper, the application of graphene technology and CNT/NR composite materials in sports equipment is discussed theoretically through the analysis of the current graphene nanofabrication technology and its application in NR, CNT and other materials. In this way, the conjugated nanofabrication process is linked to the theory in the field of sports, and its application in the field of sports is promoted. The optimal calculation model of the main raw materials of sports equipment is established, which provides a scientific theoretical basis for the development of sports equipment industry. Due to the special two-dimensional structure, large specific surface area, electrical conductivity and electrical storage properties of graphene composite materials, the application of graphene composite materials will greatly improve the capacitance of its capacitors and further improve its performance. There are some shortcomings in the research of this article, and the connection with the examples of sports equipment is sparse. In the future, specific case studies and analysis of sports equipment such as badminton rackets will be conducted.

## Data Availability

The original contributions presented in the study are included in the article/Supplementary Material, further inquiries can be directed to the corresponding author.
